# Association between formaldehyde exposure and miscarriage in Chinese women

**DOI:** 10.1097/MD.0000000000007146

**Published:** 2017-06-30

**Authors:** Wenjing Xu, Weiqiang Zhang, Xuezhen Zhang, Taowei Dong, Huiqian Zeng, Qiyun Fan

**Affiliations:** Department of Obstetrics, Guangzhou Women and Children's Medical Centre, Yuexiu, Guangzhou, Guangdong, China.

**Keywords:** Chinese women, miscarriage, plasma formaldehyde levels

## Abstract

The aim of this study was to assess whether higher plasma formaldehyde concentration existed in women diagnosed with miscarriage and whether it contributed to higher risk of miscarriage in Chinese women.

A case-control study was conducted in 118 women with a diagnosed miscarriage at the first trimester and 191 healthy women who delivered at term. Plasma levels of formaldehyde were measured by gas chromatography in conjunction with mass spectrometry after derivatization of the formaldehyde to the pentafluorophenylhydrazone and characteristics of the subjects including age, education level, occupation, family income, home decoration status, and exposure to second-hand smoke were recorded. Logistic regression analyses were performed to investigate the relationship between miscarriage and levels of formaldehyde.

Women with miscarriage were comparable to controls in terms of age, education level, occupation, family income, and home decoration status. They were, however, more likely to be exposed to second-hand smoke. Plasma levels of formaldehyde were significantly higher in women with miscarriage (0.0944 ± 0.0105 vs. 0.0239 ± 0.0032 μg/mL, *P* < .001). Multivariate logistic regression showed that higher level of formaldehyde (odds ratio [OR]: 8.06, 95% confidence interval [CI]: 4.96–13.09) and exposure to second-hand smoke (OR: 3.60, 95% CI: 1.58–8.20) were independently and significantly associated with higher risk of miscarriage.

Plasma levels of formaldehyde were significantly higher in women who were diagnosed with miscarriage than those who delivered at term and higher levels of formaldehyde was an independent risk factor for miscarriage, with higher levels being associated with higher risk of miscarriage.

## 1 Introduction

The period of pregnancy is a sensitive period during which the health status of the mother could have profound impact on the development of the foetus. In the past years, the incidence rate of miscarriage has been gradually increasing in China.^[1]^ In the Guangzhou Women and Children's medical center, there were 1837 miscarriages in the past 2 years, accounting for 9% of the total number of pregnant women who delivered in the hospital. Such increase coincides with urbanization and accompanying air pollution.

Formaldehyde is a known human carcinogen and a common source of indoor air pollution.^[2]^ It is widely used in construction, furniture, textile, medical, and chemical industries. Indoor sources of formaldehyde include building and household materials such as pressed wood, carpet, and furniture, nonelectric home cooking and heating systems, candles, and tobacco smoke.^[3]^ The concentration of formaldehyde in newly decorated rooms has been reported to exceed the China interior decoration standard and the peak value was 7 times above the standard.^[3–7]^ A Guangzhou indoor air survey has found that the highest indoor formaldehyde concentration occurred within the first year after decoration.^[6]^ But formaldehyde can be released for a long time from compound and furniture materials and become a chronic source of indoor pollution.^[8]^

Emerging evidence supports an association between formaldehyde exposure and multiple adverse health effects.^[9]^ Formaldehyde can be absorbed through the respiratory and gastrointestinal tracts and transferred from mother to foetus through the placental circulation.^[10]^ There were many experimental animal studies which indicated that maternal formaldehyde exposure could be associated with miscarriage and other adverse reproductive outcomes.^[11–15]^ However, studies in human have been very limited because of difficulties in directly measuring chronic exposures to low-level concentration of formaldehyde.^[16–22]^ The aim of this study was to assess whether higher plasma formaldehyde concentration existed in women diagnosed with miscarriage and whether it contributed to higher risk of miscarriage after considering other confounding factors. Results from this study could provide basis to support further research to identify the sources of formaldehyde and to support environmental public health policy to reduce formaldehyde exposure.

## 2 Methods

For this cross-sectional study, a consecutive cohort of 118 women who were diagnosed with miscarriage in the first trimester by ultrasound were recruited during March and April 2014 at the obstetric clinic at Guangzhou Women and Children's Medical Centre, Guangzhou, China. An age-matched group (n = 191) of pregnant women with delivery at term were recruited as controls. Controls were also matched with women with miscarriage in terms of educational level, occupation, and family income. The following characteristics were recorded: age, education level, household income, occupation (construction industry or other industries), home decoration, and smoking habit. The study protocol was approved by the Hospital's Ethics Committee and written informed consent was obtained from all patients.

Levels of formaldehyde in plasma were measured by gas chromatography (GC) in conjunction with mass spectrometry after derivatization of the formaldehyde to the pentafluorophenylhydrazone as described by Heck et al.^[23,24]^ Briefly, plasma was obtained from whole blood samples and then mixed with 2,4-dinitrophenylhydrazine. The mix was placed in 65°C for 20 minutes before cooling down and 2 mL of hexane was added for extraction of the derivatives. The specimen was then injected into the GC (Shimadzu GC 2010, Kyoto, Japan) for analysis and levels of formaldehyde were calculated against the calibration curves obtained from standard formaldehyde solution purchased from the Research Institute of the Bauru of Environment Protection of Guangzhou.

Statistical analyses were performed using IBM Statistical Package for the Social Sciences (version 20.0, SPSS Inc, Chicago, IL). Results were expressed as mean ± standard deviation or number (percentage) depending on the type of data. Comparisons of characteristics between women with miscarriage and controls were conducted using *χ*^2^ test. Binary logistic regression was used to estimate crude odds ratio (OR) for each potential risk factor and the diagnosis of miscarriage. Levels of formaldehyde were log-transformed and were first examined as a continuous variable with OR calculated as per unit change and then as a categorical variable defined by quartiles with OR calculated with the lowest quartile as the reference group. To investigate whether levels of formaldehyde was an independent risk factor for miscarriage, multivariate logistic regression was performed by adjusting other potential risk factors. All analyses were 2-tailed and a *P* < .05 was considered statistically significant.

## 3 Results

Table 1 shows characteristics of the women diagnosed with miscarriage and those who delivered at term. There was no significant difference in education level, occupation characteristics, family income per month, and home decoration status between the 2 groups. However, percentage of women who were exposed to second-hand smoke was significantly higher in women with miscarriage (59.7% vs. 40.3%, *P* < .005). The average ± SD plasma formaldehyde level was 0.0944 ± 0.0105 μg/mL in women with miscarriage, which was significantly higher than controls (0.0239 ± 0.0032 μg/mL, *P* < .001).

**Table 1:**
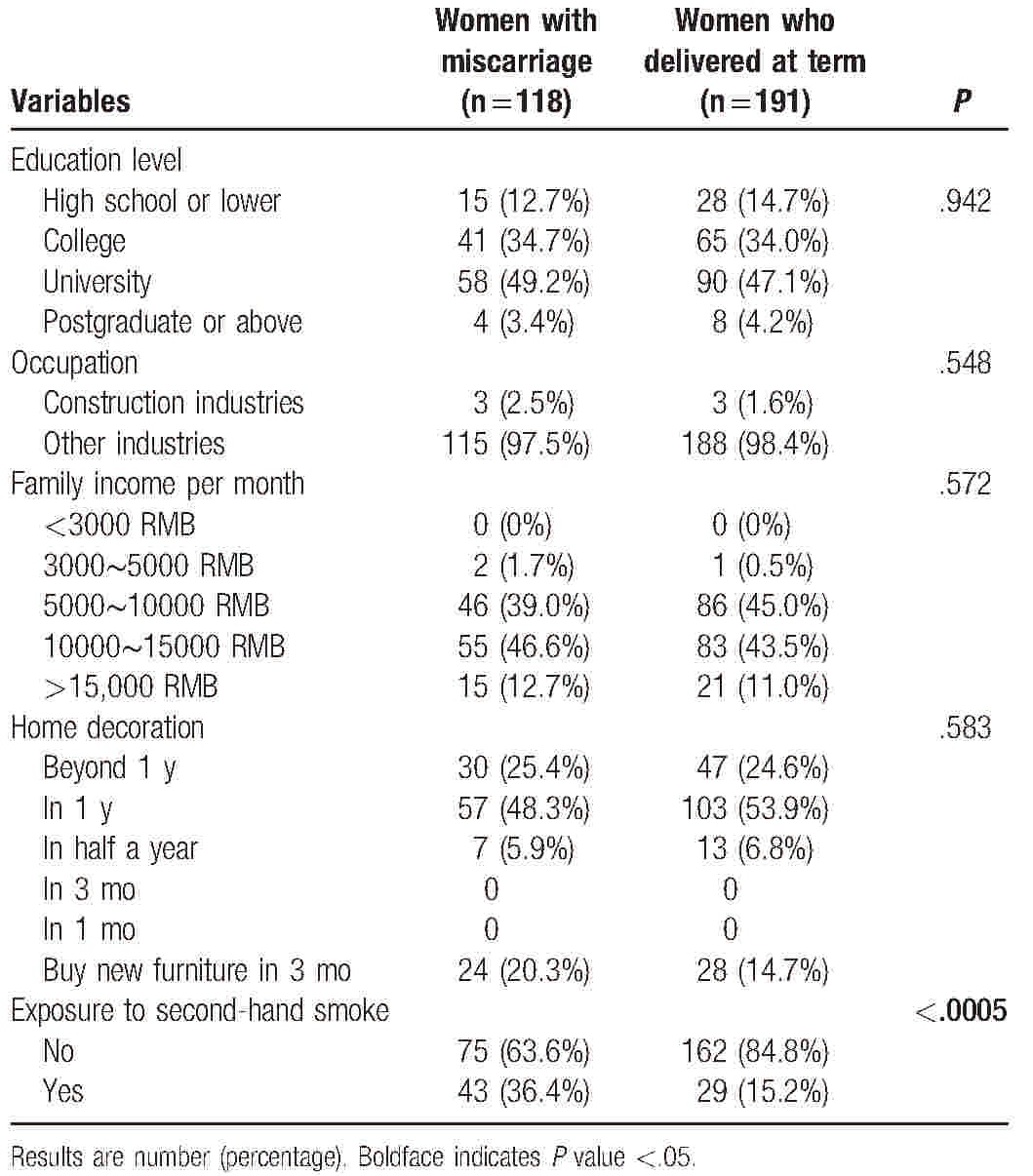
Subject characteristics.

Table 2 shows the results of logistic regression exploring potential risk factors for miscarriage in the whole cohort. In univariate analyses, education level, occupation characteristics, family income per month, and home decoration status were not significantly associated with higher risk of miscarriage. Higher plasma levels of formaldehyde (OR: 7.87, 95% confidence interval [CI]: 4.96–12.49), and exposure to second-hand smoke (OR: 3.20, 95% CI: 1.86–5.52) were significantly associated with higher risk of miscarriage (all *P* < .001). In multivariate logistic regression, after adjusting for other factors, plasma levels of formaldehyde and exposure to second-hand smoke remained significantly associated with risk of miscarriage.

**Table 2:**
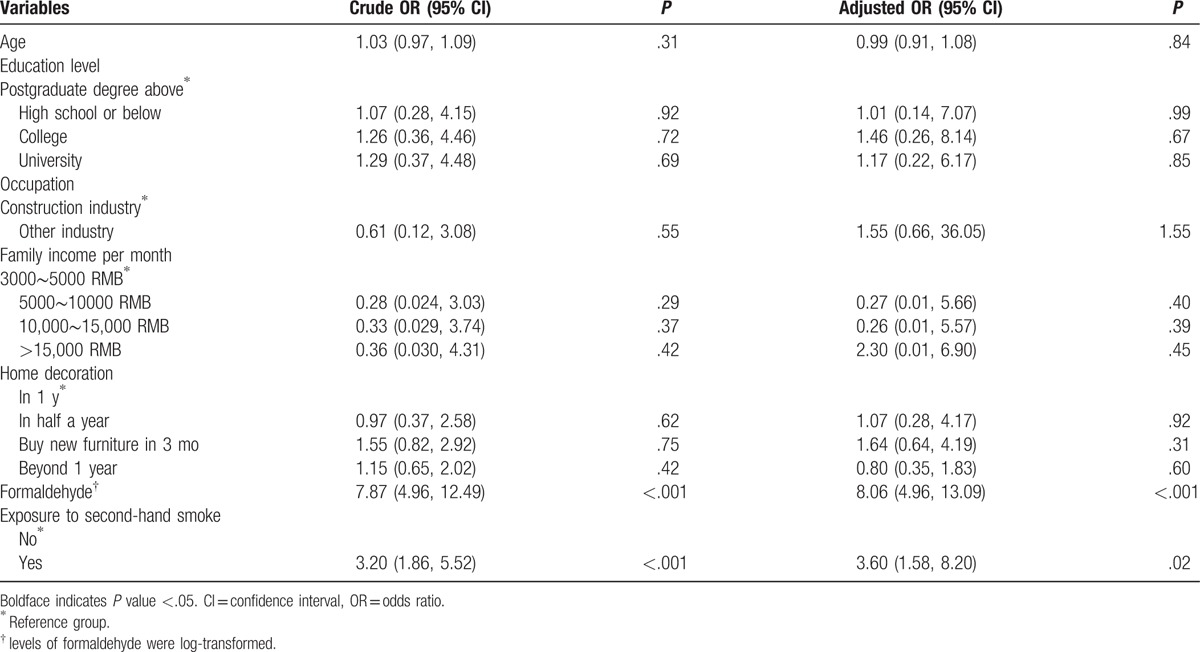
Univariate and multivariate logistic regression analyses showing ORs and 95% CI for risk factors associated with miscarriage, with levels of formaldehyde analyzed as continuous variable.

Similar results were found when levels of formaldehyde were analyzed as categorical variables. In univariate logistic regression, compared with levels of formaldehyde of the lowest quartile, ORs (95% CI) for miscarriage for second, third, and fourth quartile of formaldehyde levels were 11.45 (2.53, 51.88) (*P* = .002), 44.00 (9.97, 194.11) (*P* < .0005), and 212.67 (44.14, 1024.52) (*P* < .0005), respectively. Risk of miscarriage increased significantly with increasing levels of formaldehyde (*P* value for trend test <.0005). Table 3 showed results of multivariate logistic regression analyses with levels of formaldehyde analyzed as categorical variable. Results showed that levels of formaldehyde were an independent and significant variable associated with risk of miscarriage and higher levels indicated higher risk of miscarriage (*P* value for trend test <.0005).

**Table 3:**
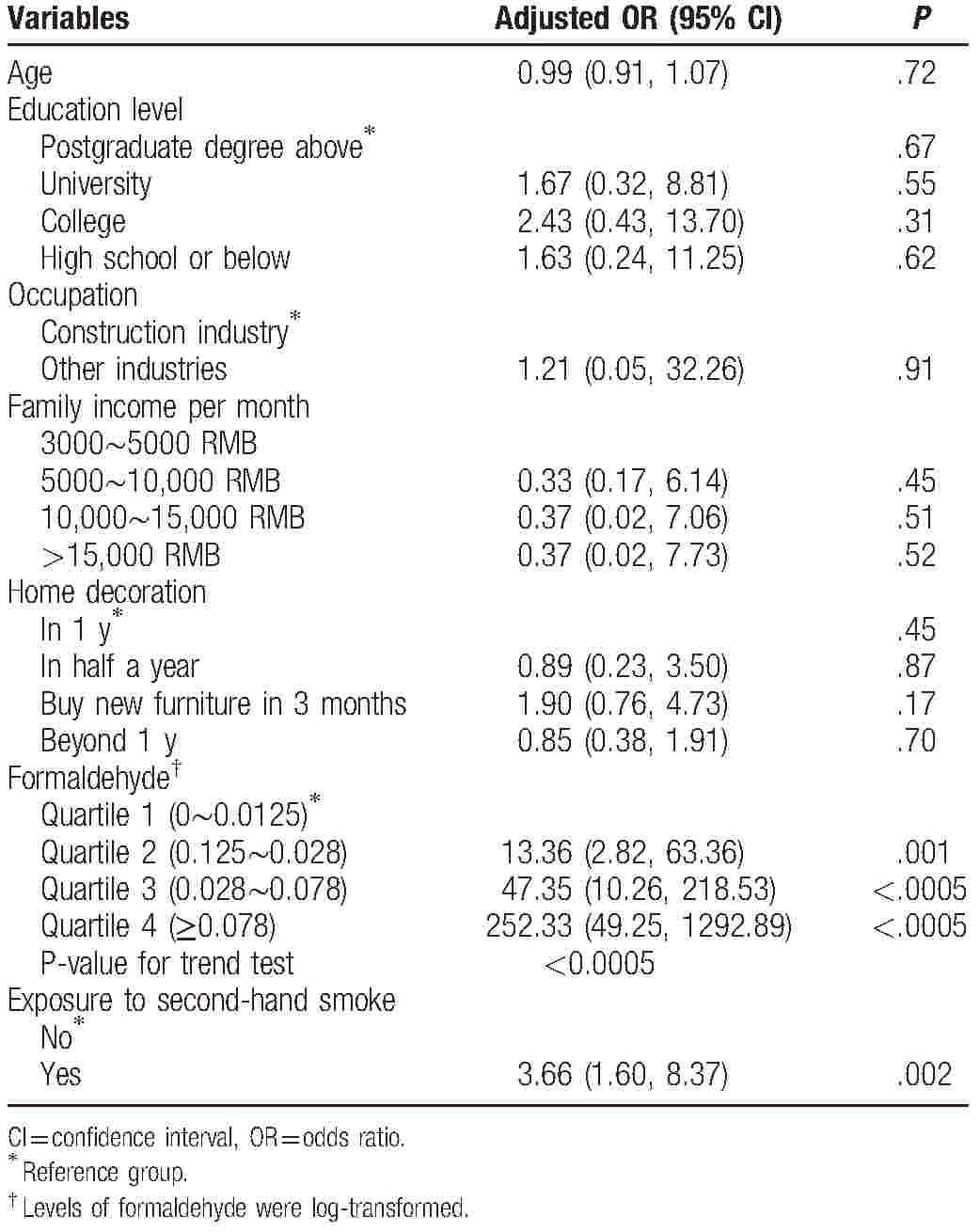
Multivariate logistic regression analyses showing ORs and 95% CI for risk factors associated with miscarriage, with levels of formaldehyde analyzed as categorical variable.

## 4 Discussion

To our knowledge, this is the first study conducted in Chinese women to investigate the risk of miscarriage and plasma levels of formaldehyde. Our results showed that plasma levels of formaldehyde were significantly higher in women who were diagnosed with miscarriage than those who delivered at term and higher levels of formaldehyde was an independent risk factor for miscarriage, with higher levels being associated with higher risk of miscarriage. Our results provide evidence of risk of development toxicity in human when exposed to indoor pollutants.

Indoors, formaldehyde is mainly emitted from building and household materials such as pressed wood, carpet, and furniture.^[7]^ In our study, although there was no significant difference in occupation and home decoration status between the groups, levels of formaldehyde were still significantly higher in women with miscarriage. Pregnancy is a particularly vulnerable period during which various physiological changes occur. It has been reported that respiratory minute ventilation, which is the volume of gas inhaled or exhaled per minute, increases with pregnancy.^[25]^ This could lead to an increase in formaldehyde inhalation. In addition, pregnant women are more likely to spend more time indoors and levels of formaldehyde is 2 10 times higher as compared to outdoor air.^[26]^ Furthermore, tobacco smoke is a potential source of formaldehyde^[27]^ and in our study, women with miscarriage were more likely to be exposed to second-hand smoke. Collectively, this would indicate an increase exposure to formaldehyde in pregnant women.

Previous studies have also showed that formaldehyde has reproductive and development toxicity to human.^[2]^ Formaldehyde is usually absorbed through the respiratory and gastrointestinal tracts and then transferred from mother to foetus through the placental circulation. Exposure to formaldehyde has been linked to higher risk of congenital anomalies, low birth weight, and premature birth.^[17,28,29]^ Higher miscarriage rate has also been reported in several previous studies performed in various groups of women. These groups included laboratory workers, cosmetologists, and wood workers. A study of 745 Swedish female university laboratory workers reported a lightly higher risk of miscarriage in women exposed to organic solvents during their first trimester (relative risk: 1.31, 95% CI: 0.89, 1.91).^[18]^ Three of the 10 (30%) women who were exposed to formaldehyde had miscarriage, compared to only 11.5% of those who did not conduct laboratory work during pregnancy (11.5%). Similar findings were also reported in laboratory workers in Finland. In this study, a significantly higher risk of miscarriage (OR: 3.5, 95% CI: 1.1–11.2) was reported in women working in laboratory who were chronically exposed to formalin.^[19]^ In United States, full-time cosmetologists who used formaldehyde-based disinfectants had a 2.1-fold (95% CI: 1.0–4.3) risk of miscarriage compared to their co-workers who did not use formaldehyde-based disinfectants.^[21]^ An increased risk of miscarriage (OR: 3.2, 95% CI: 1.2–8.3) has also been reported in female wood workers who were chronically exposed to formaldehyde.^[20]^ Results from our study add to the evidence that exposure to formaldehyde might increase the risk of miscarriage in pregnant women and higher exposure might imply a higher risk.

Our study has several limitations. First, our findings could be biased by the different gestational stages between the 2 groups. A group of pregnant women at first trimester should be included to properly delineate the relationship between formaldehyde and risk of miscarriage. Second, our study was a case-control study and could not conclude a causal relation. A prospective cohort study will be required to confirm our findings. Moreover, we did not investigate the source of formaldehyde or quantify the exposure. This could be particularly relevant to design measure to limit the exposure and reduce the risk of miscarriage.

## 5 Conclusion

In conclusion, our study showed that plasma levels of formaldehyde were significantly higher in women who were diagnosed with miscarriage than those who delivered at term and higher levels of formaldehyde was an independent risk factor for miscarriage, with higher levels being associated with higher risk of miscarriage. Our study provides evidence for the association between formaldehyde and miscarriage.

## Acknowledgments

The authors are grateful to the staff of Department of Obstetrics, Guangzhou Women and Children's Medical Centre, who had provided their support in recruiting the patients.
